# Distal weakness with respiratory insufficiency caused by the m.8344A > G “MERRF” mutation

**DOI:** 10.1016/j.nmd.2014.03.011

**Published:** 2014-06

**Authors:** Emma L. Blakely, Charlotte L. Alston, Bryan Lecky, Biswajit Chakrabarti, Gavin Falkous, Douglass M. Turnbull, Robert W. Taylor, Grainne S. Gorman

**Affiliations:** a Wellcome Trust Centre for Mitochondrial Research, Institute for Ageing and Health, The Medical School, Newcastle University, Newcastle upon Tyne NE2 2HH, UK; b The Walton Centre NHS Foundation Trust, Liverpool L9 7LJ, UK; c Aintree Chest Centre, University Hospital Aintree, Liverpool L9 7AL, UK

**Keywords:** Mitochondria, Distal myopathy, MERRF syndrome

## Abstract

The m.8344A > G mutation in the mt-tRNA^Lys^ gene, first described in myoclonic epilepsy and ragged red fibers (MERRF), accounts for approximately 80% of mutations in individuals with MERRF syndrome. Although originally described in families with a classical syndrome of myoclonus, ataxia, epilepsy and ragged red fibers in muscle biopsy, the m.8344A > G mutation is increasingly recognised to exhibit marked phenotypic heterogeneity. This paper describes the clinical, morphological and laboratory features of an unusual phenotype in a patient harboring the m.8344A > G ‘MERRF’ mutation. We present the case of a middle-aged woman with distal weakness since childhood who also had ptosis and facial weakness and who developed mid-life respiratory insufficiency necessitating non-invasive nocturnal ventilator support. Neurophysiological and acetylcholine receptor antibody analyses excluded myasthenia gravis whilst molecular genetic testing excluded myotonic dystrophy, prompting a diagnostic needle muscle biopsy. Mitochondrial histochemical abnormalities including subsarcolemmal mitochondrial accumulation (ragged-red fibers) and in excess of 90% COX-deficient fibers, was seen leading to sequencing of the mitochondrial genome in muscle. This identified the m.8344A > G mutation commonly associated with the MERRF phenotype. This case extends the evolving phenotypic spectrum of the m.8344A > G mutation and emphasizes that it may cause indolent distal weakness with respiratory insufficiency, with marked histochemical defects in muscle. Our findings support consideration of screening of this gene in cases of indolent myopathy resembling distal limb-girdle muscular dystrophy in which screening of the common genes prove negative.

## 1 Introduction

Myoclonic Epilepsy with Ragged-Red Fibers (MERRF) is a rare disorder [1], characterized by myoclonic epilepsy and cardinal histological features of mitochondrial dysfunction [2], frequently caused by the m.8344A > G mt-tRNA^Lys^ (*MTTK*) gene mutation [3]. Although the major features of MERRF include myoclonus, generalized epilepsy, ataxia and ragged red fibers in the muscle biopsy [2,3], it is increasingly recognized that phenotypic heterogeneity is common [4]. We describe a 42-year-old woman who presented with indolent, predominantly distal myopathy and respiratory insufficiency not previously associated with the m.8344A > G mutation but with significant evidence of mitochondrial histochemical abnormalities which led to a diagnosis. This study was approved and performed under the ethical guidelines issued by our institution and complied with the declaration of Helsinki.

## 2 Patient and methods

### 2.1 Case report

**Fig. 1:**
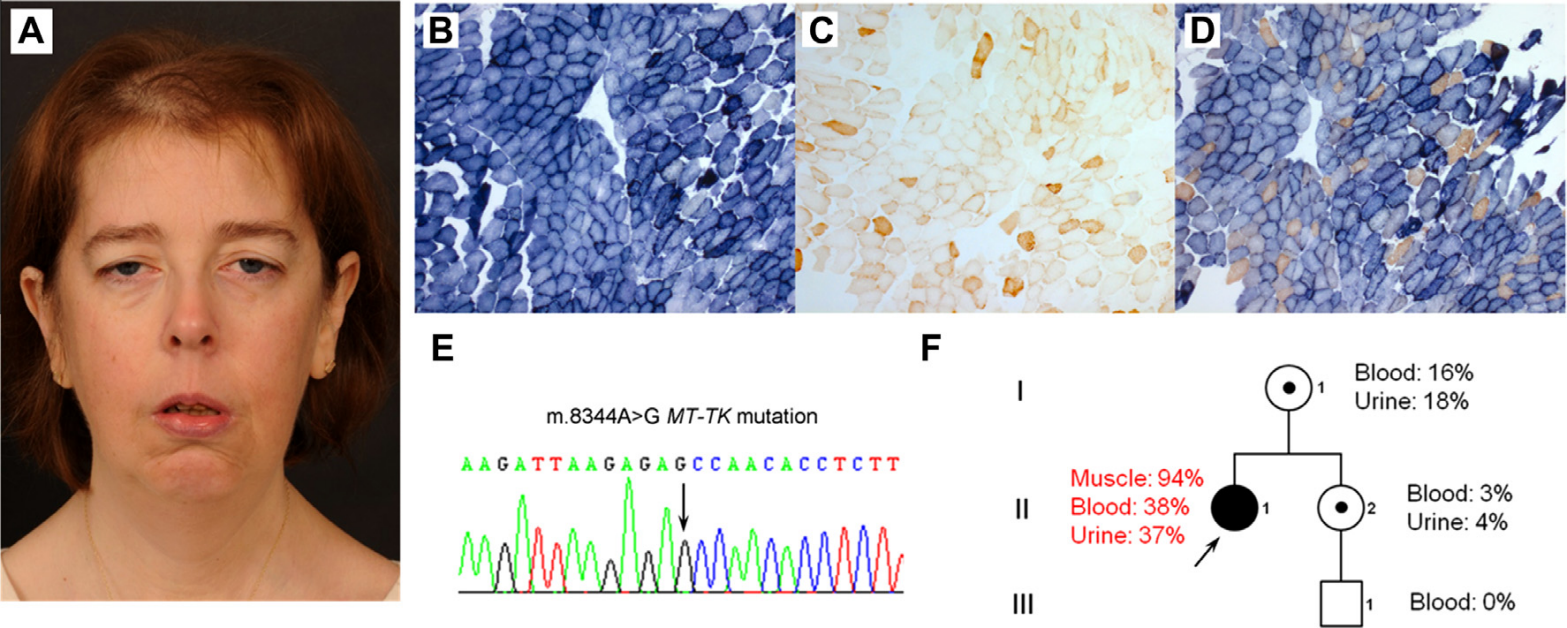
(A) Clinical features include hair thinning, bilateral ptosis, and marked facial diplegia with prominent temporalis muscle wasting, jaw weakness and mild neck flexor and extension weakness. (B) Illustrates a severe mitochondrial histochemical defect, characterized by subsarcolemmal mitochondrial accumulation (ragged-red fibers) on the SDH reaction and in excess of 90% COX-deficient fibers following COX (C) and sequential COX-SDH (D) histochemistry. (E) Sequencing of the mitochondrial genome identified the well-characterised m.8344A > G *MTTK* gene mutation which was shown to be present at very high levels of heteroplasmy in the patient’s skeletal muscle (94% mutation load) but lower levels in blood and urine by quantitative pyrosequencing. (F) Analysis of samples from the patient’s clinically-unaffected mother (16% mutation load in blood; 18% mutation load in urine) and sister (3% mutation load in blood; 4% mutation load in urine) confirmed lower levels of the m.8344A > G heteroplasmy and maternal transmission, whilst the mutation could not be detected in blood from her clinically-unaffected 14 year-old nephew.

A 42-year-old woman, born with congenital talipes equinovarus presented with slowly progressive hand weakness, reduced exercise tolerance since childhood and increasing dyspnea on exertion, migraine and treated hypothyroidism. Her mother had a cardiac pacemaker and her sister had epilepsy, but no evidence of myopathy. Her brother had ptosis, obstructive sleep apnea requiring non-invasive nocturnal ventilatory support and died of a pulmonary embolism aged 40 years. Examination showed a stature of 1.58 m, ptosis, slowed pursuit eye movements but no significant restriction, striking lower facial weakness and distal limb weakness (MRC grade 4) with relative sparing of proximal musculature, with retained tendon reflexes and normal sensation (Fig. 1A). Myotonia was not found. Creatine kinase level was normal and plasma lactic acid was raised (3.0 mmol/L normal <2.1). Nerve conduction studies and EMG, including SFEMG were normal. MRI brain imaging was not performed. Acetylcholine receptor antibody assay was negative. Cardiologic workup was normal. Forced vital capacity in the erect position was 2.0 L, and reduced to 1.5 L in the supine position, suggestive of diaphragmatic weakness. Arterial blood gases on room air showed an elevated paCO2 of 6.13 kPa (pH 7.39 and PO2 10.83) indicating hypercapnia. Overnight oximetry showed that she spent 38 min below 90% oxygen saturation and 26 min below 88% oxygen saturation, indicative of hypoventilation. Non-invasive nocturnal ventilatory support was commenced. Molecular genetic testing excluded myotonic dystrophy prompting a diagnostic needle muscle biopsy.

### 2.2 Histopathology and molecular genetic studies

Standard histopathological analysis of muscle was undertaken. Total genomic DNA was extracted from blood, urinary epithelium and skeletal muscle using standard procedures. Mitochondrial DNA rearrangements were investigated in muscle by long-range PCR whilst direct sequencing of the entire mitochondrial genome was undertaken as previously described [5]. Amplified PCR products were sequenced using BigDye® Terminator v3.1 chemistries (Applied Biosystems) and compared to the revised Cambridge reference sequence (GenBank Accession number NC_012920.1). Quantification of m.8344A > G mutation load by pyrosequencing [6], was performed using mutation-specific primers (details available on request).

## 3 Results

Muscle biopsy analysis showed remarkable mitochondrial histochemical abnormalities characterized by subsarcolemmal mitochondrial accumulation (ragged-blue fibers) on the SDH reaction (Fig. 1B) and in excess of 90% COX-deficient fibers (Fig. 1C and D), prompting genetic studies to investigate a likely mitochondrial etiology. Long-range PCR across the major mtDNA arc revealed no evidence of mtDNA rearrangements, and a screen for the m.3243A > G mutation was negative. Sequencing of the mitochondrial genome in muscle identified the well-characterised m.8344A > G mutation commonly associated with the MERRF phenotype (Fig. 1E). Quantitative pyrosequencing confirmed the m.8344A > G mutation to be present at very high levels of heteroplasmy in the patient’s skeletal muscle (94% mutation load) but lower levels in blood (38%) and urine (37%) (Fig. 1F). Analysis of samples from the patient’s clinically unaffected mother (16% mutation load in blood; 18% mutation load in urine) and sister (3% mutation load in blood; 4% mutation load in urine) confirmed lower levels of m.8344A > G heteroplasmy and maternal transmission, whilst the mutation could not be detected in blood from her clinically unaffected 14 year-old nephew. Unfortunately, tissue samples were unavailable from her deceased brother or maternal grandmother for mutational analysis.

## 4 Discussion

We report a 42-year-old woman with exceptional clinical and histological features of the m.8344A > G mutation; very high levels of the mutation were detected in skeletal muscle, associated with ptosis, marked facial and distal muscle weakness, respiratory insufficiency and severe COX deficiency. To date, over 300 patients with MERRF syndrome due to the m.8344A > G mutation have been described [4], and more than 40 m.8344A > G patients have been reviewed at our centre; none appear to share this unusual clinical and histochemical phenotype. Moreover, facial weakness is a relatively uncommon muscle manifestation of mitochondrial disorders. The marked mitochondrial histochemical abnormalities were suggestive of a pathogenic mtDNA mutation, exhibiting high mutation threshold. However, due to the severity of the histochemical defect with in excess of 90% COX-deficient fibers in the muscle biopsy, and with a phenotype strongly suggestive of a limb girdle muscular dystrophy initial genetic testing was directed to whole mtDNA genome sequencing in preference to targeted m.8344A > G mutation analysis. Biopsy findings, combined with the conspicuous discrepancy in the segregation of m.8344 > G mutation load between tissues, we suggest, accounts for the myopathic phenotype although potential *cis*-acting modifiers were not evaluated [7].

In conclusion, this case emphasizes that the m.8344A > G mutation can cause indolent distal weakness with respiratory insufficiency, with marked histochemical defects in muscle and extends the evolving phenotypic spectrum attributable to the m.8344A > G “MERRF” mutation.

## Authors’ contribution

**ELB:** Acquisition and interpretation of data, critical revision of the manuscript for important intellectual content.

**CLA:** Acquisition and interpretation of data, critical revision of the manuscript for important intellectual content.

**BL:** Study concept and design, acquisition and interpretation of data, critical revision of the manuscript for important intellectual content.

**GF:** Acquisition and interpretation of data.

**DMT:** Analysis and interpretation of data, critical revision of the manuscript for important intellectual content.

**RWT:** Study concept and design, analysis and interpretation of data, critical revision of the manuscript for important intellectual content.

**GSG:** Study concept and design, analysis and interpretation of data, critical revision of the manuscript for important intellectual content, study supervision and co-ordination.

## Funding

This work was supported by the Wellcome Trust [096919Z/11/Z and 074454/Z/04/Z to D.M.T., R.W.T.]; the Medical Research Council [G0601943 and G0800674 to D.M.T., R.W.T.]; the UK National Institute for Health Research Biomedical Research Centre for Ageing and Age-related Diseases award to Newcastle upon Tyne Hospitals NHS Foundation Trust [to D.M.T., G.S.G.] and the UK NHS Specialized Services and Newcastle upon Tyne Hospitals NHS Foundation Trust that support the ‘Rare Mitochondrial Disorders of Adults and Children’ Diagnostic Service [http://www.newcastle-mitochondria.com].

## Permission to publish

Thank you to the patient, who has kindly given permission for publication of this case report including clinical image.
